# Design strategies to influence health behaviour and mental wellbeing in the urban setting

**DOI:** 10.1093/eurpub/ckac129.397

**Published:** 2022-10-25

**Authors:** J Rehn, H Müller, A Wasmer, E Chrysikou

**Affiliations:** 1 Design Research, Darmstadt University of Applied Sciences, Darmstadt, Germany; 2 Social and Cultural Sciences and Social Work, Darmstadt University of Applied Sciences, Darmstadt, Germany; 3 Civil Engineering, Darmstadt University of Applied Sciences, Darmstadt, Germany; 4 Bartlett Real Estate Institute, University College London, London, UK

## Abstract

The design of objects, spaces and systems can have a profound influence on the behaviours as well as emotional and cognitive states of the people confronted with it. With regards to health behaviour, elaborating on Schwarzer's HAPA model (1992), the Design Model for Health Behaviour Change - DMHBC (Rehn, 2018) proposes the use of the built environment to act as situative barriers or opportunities to change health behaviours and overall health promoting mindsets. Regarding urban space and mental health, most environmental stimuli and related behavioural patterns focus on consumption (e.g. retail stores) or daily routines (e.g. commuting). Using objects such as specifically designed furniture, installations and other elements to act as perceived affordances and stimuli can affect both cognitive-emotional states as well as specific behavioural responses. For instance, based on the research on mindfulness, drawing people's attention towards their own bodily sensations (e.g. breath) by playful interactive installations or information signs can increase feelings of calm, appreciation and contentment. The same applies to design interventions that guide one's view towards otherwise overlooked urban features (e.g. natural scenery). While mindfulness and relaxation are powerful techniques for increasing mental health, many other approaches (such as physical activity, social interactions etc.) can be found to have similar benefits. In fact, the orchestrated combination of various forms of stimuli might prove to be more effective than the sum of the individual interventions as they create a subsequent chain of stimuli that form a coherent experience. This approach poses particular potentials to foster mental health in vulnerable groups that usually suffer most from urban environmental risk factors. Thus, providing public and open access stimuli and affordances in this way, can have a significant effect on overall urban public health and reduce social inequalities at the same time.

